# Incidence, risk factors, and outcomes of early postoperative hyperglycemia in surgical patients: a protocol for a systematic review and meta-analysis

**DOI:** 10.1186/s13643-020-01416-4

**Published:** 2020-07-13

**Authors:** Paddy Ssentongo, Joseph A. Lewcun, Anna E. Ssentongo, David I. Soybel

**Affiliations:** 1 Department of Public Health Sciences, Penn State Hershey College of Medicine and Milton S. Hershey Medical Center, 500 University Drive, Hershey, PA 17033 USA; 2 Center for Neural Engineering, Department of Engineering, Science and Mechanics, The Pennsylvania State University, University Park, PA 16802 USA; 3 Department of Surgery, Penn State Hershey College of Medicine and Milton S. Hershey Medical Center, Hershey, PA 17033 USA; 4 Penn State Hershey College of Medicine and Milton S. Hershey Medical Center, Hershey, PA USA

**Keywords:** Early postoperative hyperglycemia, Risk factors, Mortality, Meta-analysis

## Abstract

**Background:**

Early postoperative hyperglycemia (POHG) is common and associated with poor postoperative outcomes. Currently, there is no systematic review and meta-analysis that addresses the knowledge gap of the incidence of POHG in surgical patients and that explores the associated risk factors and complications. The objective of this study will be to estimate the pooled incidence, risk factors, and clinical outcomes of early postoperative hyperglycemia in men and women globally.

**Methods:**

We designed and registered a study protocol for a systematic review and meta-analysis of studies reporting the incidence of postoperative hyperglycemia (POHG). We will search PubMed (MEDLINE), Scopus, Web of Science, EMBASE, Cochrane Library, OVID (HEALTH STAR), OVID (MEDLINE), and Joana Briggs Institute EBF Database (from inception onwards). Randomized controlled trials and observational cohort studies reporting the incidence of POHG and conducted in surgical patients will be included. No age, geographical location, study design, or language limits will be applied. The primary outcome will be the incidence of POHG. Secondary outcomes will be risk factors and clinical outcomes of POHG. Two reviewers will independently screen citations, full text articles, and abstract data, extract data, and evaluate the quality and bias of included studies. Discrepancies will be resolved through discussion or consultation with a third researcher. The risk of bias and study methodological quality of included studies will be evaluated by the appropriate Cochrane risk of bias tool for randomized trials and Newcastle-Ottawa Scale for cohort studies. If feasible, we will conduct random effects meta-analysis with a logit transformation of proportions. We will report the probability of postoperative hyperglycemia as a measure of incidence rate, relative risk ratios (RR), and 95% confidence intervals to report the effects of the risk factors and postoperative outcomes. Additional analyses will be conducted to explore the potential sources of heterogeneity (e.g., age, gender, geographical location, publication year, comorbidities, type of surgical procedure). The Egger test and funnel plots will be used to assess small study effects (publication bias).

**Discussion:**

This systematic review and meta-analysis will identify, evaluate, and integrate the evidence on the incidence, risk factors, and outcomes of early POHG in surgical patients. The results of this study can be used to identify populations which may be at particular risk for POHG. Future studies which use this information to better guide post-operative glycemic control in surgical patients could be considered.

**Systematic review registration:**

PROSPERO registration number CRD42020167138

## Background

Early postoperative hyperglycemia (POHG) is recognized as a major cause of delays in convalescence, higher rates of complications, and increased costs of care [1–4]. As a form of stress hyperglycemia, POHG usually resolves spontaneously in the days following operation when the counter-regulatory hormones and pro-inflammatory cytokines return to normal levels [5]. However, it is associated with several adverse outcomes and complications including higher mortality, sepsis, surgical site infection, pneumonia, and prolonged hospitalization [2, 6]. The incidence of stress hyperglycemia is more likely in patients with a preoperative diagnosis of type 2 diabetes (T2D) and impaired glucose metabolism and control [3]. Strikingly, POHG has been observed among patients who are not known to have T2D [1, 2, 7–10]. In such a cohort of patents, adverse outcomes occurring in association with POHG may be as or more consequential [1, 3, 7, 9] than in patients with a diagnosis of T2D. Moreover, a recent study has provided seemingly paradoxical evidence that higher levels of hemoglobin A1C may be associated with a lower likelihood of post-operative complications and readmissions [3]. These observations suggest a complex set of relationships between pre-operative glucose control, early post-operative dysglycemia, and outcomes after surgical procedures. Furthermore, they highlight an unmet need to delineate independent risk factors of POHG and its associated complications.

Among patients being cared for in surgical units, the incidence of POHG is quite high, depending on the attributes of the institution and its catchment area, the nature of the illnesses being treated, patient comorbidities, and the specific classes of surgical procedures. The incidence of POHG ranges from 22% in vascular surgeries [6] to 77% in cardiac surgeries [11] and from 16% in non-diabetics [12] to 24% in diabetics [13]. Perioperative and intensive care unit blood glucose control and management without major glycemic variation have been shown to improve outcomes [14–16].

A 2013 meta-analysis by Sathya et al. found that in patients with T2D, a moderate perioperative glycemic target (150–200 mg/dL) was associated with a reduction in postoperative mortality [17]. A second meta-analysis conducted by Haga et al. in 2012 suggested that tight glycemic control after cardiac surgery was associated with better post-operative outcomes [18]. However, to our knowledge, there is no systematic review and meta-analysis that addresses the knowledge gap of the incidence of POHG in surgical patients and that explores the associated risk factors and complications.

## Objectives

The objective of this study will be to conduct a systematic review and meta-analysis to ascertain the incidence of POHG and to delineate the associated risk factors and poor postoperative outcomes. Specific aims are to determine:

(i) What is the global incidence of POHG in surgical patients?
(ii) What are the risk factors (T2D, obesity, American Society of Anesthesiologists classification, sex, operative time) associated with POHG in surgical patients?
(iii) What are the clinical outcomes (such as mortality, infections rates, length of hospital stay, surgical site occurrence) associated with POHG in surgical patients?

## Methods

The present protocol has been registered within PROSPERO (registration number: CRD42020167138). The present study protocol is being reported in accordance with the reporting guidance provided in the Preferred Reporting Items for Systematic Reviews and Meta-Analyses Protocols (PRISMA-P) statement [19, 20] (see PRISMA-P checklist in Additional file 1).

### Eligibility criteria

Studies will be selected according to the following criteria: participants, condition or outcome(s) of interest, study design, and context.

1. *Participants (population)*: We will include studies involving children, adolescents, and adult patients undergoing surgery (regardless of age or sex). Studies not conducted in humans will be excluded.
2. *Condition or outcome(s) of interest*: The primary outcome will be the incidence of postoperative hyperglycemia indicating the rate of new (or newly diagnosed) cases of postoperative hyperglycemia. It is generally reported as the number of new cases occurring within a period of time (e.g., per month, per year) or as a fraction of the population at risk of developing the outcome (e.g., new cases per 1,000 or 10,000). We will use author-reported definitions (according to accepted diagnostic criteria). For example, early postoperative hyperglycemia will be defined as a high blood glucose within 48 h postoperatively. The definition of “high blood glucose” will be study-specific. We anticipate a vast majority of published studies will define postoperative hyperglycemia as a blood glucose of 140 mg/dL and above. Secondary outcomes will be the risk factors associated with postoperative hyperglycemia and clinical outcomes associated with postoperative hyperglycemia.
3. *Study design and context*: Eligible studies will be randomized controlled trials and observational cohort (prospective or retrospective) studies reporting outcome data and conducted in a wide range of surgical patients. We will exclude cross-sectional studies, case-control studies, case series, and case reports. Reviews and commentaries will be excluded, as well as studies that do not report the incidence of POHG. No limitations will be imposed on study conduct period and language of publication.

### Information sources and search strategy

#### Database searches

The primary source of literature will be a structured search of the following databases: PubMed (MEDLINE), Scopus, EMBASE, Cochrane Library, OVID (HEALTH STAR), OVID (MEDLINE), Joana Briggs Institute EBF Database, and Web of Science. The secondary source of potentially relevant material will be a search of the grey or difficult to locate literature, including Google Scholar. We will use a snowballing method (hand-searching of reference lists) to include a search the citation lists of included papers. This will be accomplished by using the “cited by” tool in Google Scholar. Efforts will be made to contact authors of ongoing studies and in-press literature for information regarding additional studies or missing data.

#### Search strategy and terms

Our keyword search will be based on Medical Subject Headings (MeSH) and text words. The surgical care filter will contain the following MeSH terms: “Postoperative Period,” OR “Perioperative Period,” OR “Surgical Procedures, Operative.” The early postoperative hyperglycemia contained MeSH terms “Hyperglycemia” OR “Blood Glucose” OR “Hypoglycemic agents” with text words “blood glucose” OR “glycemic control” OR “insulin.” This search strategy will be further adapted and tailored for use with each database, using Boolean operators (OR/AND), truncations, proximity operators, and Medical Subject Heading, as appropriate for each database.

#### Study selection and data extraction

Two review team members (JAL, AES) will independently screen all studies identified from the literature search in two stages. In the first stage, the two reviewers (JAL, AES) will independently screen titles and abstracts based on the eligibility criteria outlined above. They will document, with reasons, the studies excluded from the review. The citations will be downloaded into the Endnote software and will exclude duplicate articles. In the second stage, full-text versions of selected abstracts will be downloaded/retrieved and examined in detail by the two reviewers (JAL, AES) for eligibility. They will extract data from eligible papers identified during the abstract screening step. In the event of disagreement, the two authors will confer and discuss with each other and, if necessary, a third review author (PS) to reach consensus. References of all considered articles will be hand-searched to identify any relevant report missed in the search strategy. When abstracts and subsequently included papers are not available in English, translators will be sought. Using the format of the validated standard data extraction form [21], we will extract the following information: first author, country in which the study was conducted, year of publication, study period, research methodology, total sample size using study level median age, study level gender proportions, proportion of type 2 diabetes, mean body mass index, American Society of Anesthesiologists and type of surgical procedure and study limitations. Data will be extracted independently by two authors (JAL, AES). In case of missing data, one attempt will be made to contact the corresponding authors of studies by email. If the author fails to provide additional information, a decision will be made as to whether to include the study in the final review. A flow chart showing the studies included and excluded at each stage of the study selection process will be provided.

#### Assessment of methodological quality of the papers

Two authors (JAL, AES) will independently assess the quality of the papers included in the review. Assessment of methodological quality will be conducted using the Cochrane risk of bias tool for randomized trials and the Newcastle-Ottawa Scale (NOS). NOS is a validated tool for assessing quantitative cross-sectional, case-control, and cohort studies [22]. Scores between 7 and the maximum score of 9 will be defined as high quality; scores between 4 and 6 will be defined as intermediate quality; and scores between 1 and 3 will be defined as low quality. Discrepancies in scoring will be resolved by discussion with a third author (PS). Studies will be included regardless of the risk of bias and quality scores, but sensitivity analysis will be conducted to ascertain the impact of their inclusion.

#### Data synthesis

We will synthetize primary studies to explore heterogeneity descriptively such as structured narratives or summary tables, measures of prevalence, and incidence of POHG. Different patients and different studies are unavoidably heterogeneous. Although no widely accepted quantitative measure exists to grade clinical heterogeneity [23], we will not do meta-analysis if the clinical and surgical procedures are too different. The degree of difference will be discussed with the board-certified general surgeon (DIS) who will decide whether the clinical and surgical variation of the studies is too high to carry out a meta-analysis. If data are appropriate for quantitative synthesis of primary and secondary outcomes, we will conduct random-effects meta-analysis of incidence data. The data from each paper (e.g., study characteristics, outcomes and findings) will be used to build evidence tables of an overall description of included studies. Incidence estimates of postoperative hyperglycemia will be presented as new cases per 1000 along with 95% confidence intervals [24]. Relative risk ratios (RR) or odds ratios (OR) with 95% confidence intervals will be used to report the association of postoperative hyperglycemia with the risk factors and postoperative outcomes. If feasible and appropriate, data points from primary studies will be used to perform random effects meta-analyses. Since heterogeneity is expected a priori, we will estimate the pooled incidence and its 95% confidence interval using the random-effects model with logit transformation and back transformation. The random-effects model assumes the study estimates follow a normal distribution, considering both within-study and between-study variations. Forest plots will be used to visualize the extent of heterogeneity among studies. We will quantify statistical heterogeneity by estimating the variance between studies using *I*^2^ statistic. The *I*^2^ is the proportion of variation in prevalence estimates that is due to genuine variation in prevalence rather than sampling (random) error. *I*^2^ ranges between 0 and 100% (with values of 0–25% and 75–100% taken to indicate low and considerable heterogeneity, respectively). We will also report Tau^2^ and Cochran *Q* test with a *P* value of < 0.05 considered statistically significant (heterogeneity).

#### Additional analyses

If sufficient studies are identified and data points are available, potential sources of heterogeneity will be investigated further by subgroup or meta-regression analyses according to baseline characteristics and methodological covariates [5]. We plan to conduct subgroup and/or meta-regression analyses by geographical location (e.g., region and/or country), age (e.g., median), gender (e.g., proportion of women), year of study conduct, comorbidities (e.g., proportion of type 2 diabetes, mean body mass index, American Society of Anesthesiologists status), study design (e.g., randomized controlled trials vs cohort studies), and type of surgical procedure.

#### Meta-bias

The Egger test and funnel plots will be used to assess publication bias, with the results considered to indicate potential small study effects when *P* values are < 0.10. In the presence of asymmetrical funnel plots and significant Egger’s test, trim and fill analyses will be conducted, and adjusted effect sizes will be reported. In addition, influence analysis will be performed. The analysis excludes and replaces one study at a time (leave-one-out method) from the meta-analysis and calculating the pooled effect size for the remaining studies [17, 18]. A second sensitivity analysis will be performed by subgroup analysis between high-quality and medium/low-quality studies.

#### Software considerations

We will use the metaprop function of the meta-package in R Statistical Software for analysis [24].

#### Confidence in cumulative evidence

Strength of evidence will be assessed using the GRADE (Grading of Recommendations, Assessment, Development and Evaluations) framework using four levels of quality of evidence: very low, low, moderate, and high. We will use the following domains of GRADE: risk of bias, imprecision, inconsistency, indirectness, and publication bias [25]. We will report the overall strength of evidence of the outcome of interest.

#### Patient and public involvement

Patients were not involved in the development of this systematic review protocol.

## Discussion

The systematic review and meta-analysis of observational data presented in this protocol will identify, collect, and evaluate the existing knowledge underlying the incidence, risk factors, and clinical outcomes associated with perioperative hyperglycemia in surgical patients. We are not aware of another systematic review and meta-analysis addressing this specific issue. In our opinion, this systematic review will help to establish the extent of the epidemiological evidence on this topic, in a reproducible and rigorous way. The proposed systematic review and meta-analysis will be reported in accordance with the reporting guidance provided in the Preferred Reporting Items for Systematic Reviews and Meta-analyses (PRISMA) statement [26] and the Meta-analysis Of Observational Studies in Epidemiology (MOOSE) reporting guideline [27]. Any amendments made to this protocol when conducting the study will be outlined in PROSPERO and reported in the final manuscript. Results will be disseminated through conference presentations and publication in a peer-reviewed journal. Major limitation is the inconsistence in the reporting of POHG at the study level. Such inconsistences may lead to lower or higher pooled prevalence of POHG. In addition, we anticipate high heterogeneity in the surgical procedure. At the reviewer level, we anticipate facing difficulty of choosing the risk factors of POHG due to the various reporting of these factors from individual studies. We will first conduct qualitative synthesis before carrying out meta-analysis. If we find a very high degree of clinical and methodological heterogeneity, we will not pool the results but will instead summarize the results qualitatively by using tables and figures. If we end up conducting a meta-analysis, we will however mitigate the heterogeneity by conducting subgroup analysis and meta-regression. The results of this systematic review and meta-analysis will be presented at conferences and published in a peer-review journal. The results will guide future population-specific interventions and may improve perioperative assessment and management of surgical patients, especially those in subgroups that could be at heightened risk.

## Supplementary information

**Additional file 1.** The Preferred Reporting Items for Systematic Reviews and Meta-Analyses for Protocols (PRISMA-P) checklist was used in this protocol.

## Data Availability

Not applicable

## Abbreviations

POHG: Postoperative hyperglycemia
PRISMA-P: Preferred Reporting Items for Systematic reviews and Meta-Analysis protocol
